# CCAAT/Enhancer Binding Protein ***β*** in relation to ER Stress, Inflammation, and Metabolic Disturbances

**DOI:** 10.1155/2015/324815

**Published:** 2015-01-28

**Authors:** Sophie E. van der Krieken, Herman E. Popeijus, Ronald P. Mensink, Jogchum Plat

**Affiliations:** Department of Human Biology, NUTRIM School for Nutrition, Toxicology and Metabolism, Maastricht University, Universiteitssingel 50, P.O. Box 616, 6200 MD Maastricht, Netherlands

## Abstract

The prevalence of the metabolic syndrome and underlying metabolic disturbances increase rapidly in developed countries. Various molecular targets are currently under investigation to unravel the molecular mechanisms that cause these disturbances. This is done in attempt to counter or prevent the negative health consequences of the metabolic disturbances. Here, we reviewed the current knowledge on the role of C/EBP-*β* in these metabolic disturbances. C/EBP-*β* deletion in mice resulted in downregulation of hepatic lipogenic genes and increased expression of *β*-oxidation genes in brown adipose tissue. Furthermore, C/EBP-*β* is important in the differentiation and maturation of adipocytes and is increased during ER stress and proinflammatory conditions. So far, studies were only conducted in animals and in cell systems. The results found that C/EBP-*β* is an important transcription factor within the metabolic disturbances of the metabolic system. Therefore, it is interesting to examine the potential role of C/EBP-*β* at molecular and physiological level in humans.

## 1. Introduction

The prevalence of obesity is increasing worldwide. This is worrisome regarding the known association of obesity with the development of the metabolic syndrome. The metabolic syndrome comprises a cluster of disturbances in metabolism such as an impaired glucose and lipid metabolism, high blood pressure, dyslipidemia, and a proinflammatory state [1]. Eventually, these disturbances might result in the development of type II diabetes mellitus and cardiovascular diseases [2].

Nowadays it is acknowledged that adipose tissue is a highly endocrine organ that secretes several adipokines, including a variety of proinflammatory cytokines [3]. As a consequence, there is a constant exposure to low-grade systemic inflammation. Typically, elevated concentrations of C-reactive protein, various cytokines, prothrombotic molecules, and adhesion molecules are present in this inflammatory state [4–6] which play an essential role in the development of atherosclerosis and insulin resistance [7, 8].

It is intriguing to consider the variety and number of processes that are differentially regulated between metabolic syndrome patients and healthy subjects. Many of these processes are regulated at the level of gene expression, which is controlled by downstream processes and factors, such as RNA processing, mRNA translation, mRNA degradation, and interaction with other proteins. Probably the most influential step in regulating gene expression is the rate of transcriptional regulation. Transcription factors are able to interact with regulatory sequences of target genes, thereby influencing their expression level and consequently influencing metabolism in a direct and/or indirect manner [9].

Next to transcription factors such as PPARs, NF*κ*B, and chREBP [10–12], there are strong indications that also the transcription factor CCAAT/enhancer binding protein (C/EBP-*β*) is involved in processes related to the metabolic syndrome. C/EBP-*β* knockout mice fed a high-fat diet showed a decreased fat mass, decreased serum triacylglycerols and cholesterol concentrations, and lower hepatic triacylglycerol concentrations compared to their wild type littermates [13]. Moreover, expression of hepatic lipogenic genes was downregulated, while the expression of *β*-oxidation genes in brown adipose tissue was increased [13]. These effects are in line with earlier observations that the beta variant of C/EBP is important in the differentiation and maturation of adipocytes [13]. Finally, since C/EBP-*β* is activated in proinflammatory conditions [14], there might also be a link between C/EBP-*β* and low-grade systemic inflammation. Therefore, in this review, we will focus on the influence of CCAAT/enhancer binding protein *β* and its isoforms on metabolic disturbances related to the metabolic syndrome.

## 2. CCAAT/Enhancer Binding Proteins

### 2.1. The C/EBP Family of Transcription Factors

CCAAT/enhancer binding proteins (C/EBPs) are a six-member family (*α* to *ζ*) of transcription factors. They are involved in the regulation and expression of numerous genes. C/EBPs affect gene expression by binding to a DNA binding site (consensus sequence “CCAAT”), which is present in many gene promoter and enhancer regions. All members of the C/EBP family contain a basic leucine zipper (bZIP) domain at the carboxyl-terminus (C-terminus), which is involved in dimerization and binding to the DNA [9]. Specifically, in all isoforms of C/EBP, an extension of the zipper dimerization domain, the tail sequence, acts as a motif for protein-protein interactions. For a detailed description of the structure of CCAAT/enhancer binding family members, we refer to two earlier reviews [9, 15].

### 2.2. Tissue Specific C/EBP Expression of Variants *α* to *ζ*

C/EBP protein variants are differentially expressed in various tissues. C/EBP-*α* is expressed predominantly in liver and adipose tissue and at lower level in lungs, intestine, adrenal gland, placenta, and peripheral-blood mononuclear cells [16–19]. C/EBP-*β* is highly expressed in the intestine, liver, kidney, lungs, spleen, adipose tissue, pancreatic *β*-cells [20], and monocytes and granulocytes [9, 16, 17, 21–26]. The expression of the *δ*-member of the C/EBP family is restricted to adipose tissue, intestine, and lungs, whereas C/EBP-*ε* expression is found primarily in myeloid and lymphoid cells [9, 18, 27, 28]. Finally, C/EBP-*γ* and C/EBP-*ζ* are ubiquitously expressed in most tissues [9, 29, 30].

### 2.3. A Focus on the CCAAT/Enhancer Binding Proteins Family Member C/EBP-*β*

Given the large number of publications describing a link between the *β* isoform of C/EBP and one or more characteristics of the metabolic syndrome, we decided to focus in more detail on the C/EBP-*β* isoform. In the literature, C/EBP-*β* is known under various names: NF-IL6 (nuclear factor for IL-6), TCF5 (transcription factor 5), LAP (liver-enriched activator protein), IL-6DBP (IL-6 dependent DNA binding protein), CRP2 (C/EBP-related protein 2), AGP/EBP (alpha-1-acid glycoprotein enhancer binding protein), NF-M, SF-B (silencer factor), or ApC/EBP [9]. C/EBP-*β* forms hetero- and homodimers, thereby altering its preferential DNA binding to initiate transcription of target genes involved in various cellular processes [9, 14, 19, 31–34].

### 2.4. C/EBP-*β* Isoforms

**Figure 1:**
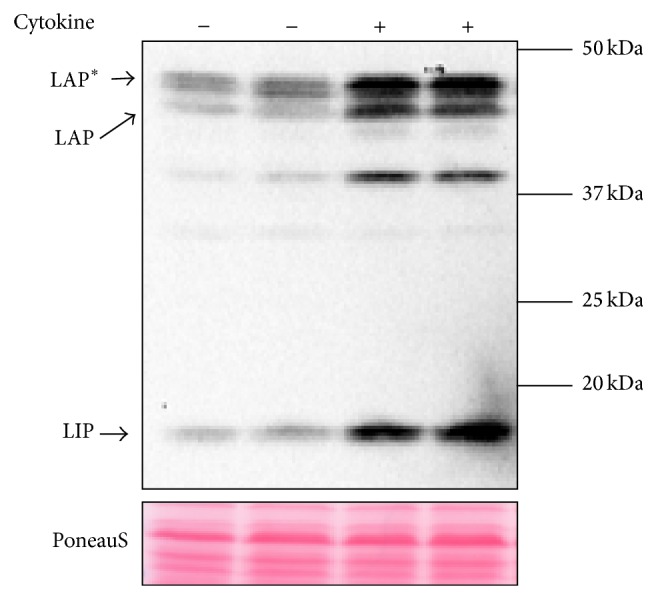
Human C/EBP-*β* protein expression in human liver carcinoma cells (HepG2 cells) under normal and after inflammation induced C/EBP-*β* activation by the addition of a cytokine cocktail for 48 h (IL-6, IL-1*β*, and TNF-*α*), detected by western blotting. Human C/EBP-*β* isoforms LAP^*^, LAP, and LIP are indicated using the arrows (note: they run at different size as the mouse isoforms (Santa Cruz Biotechnology, C/EBP-*β* (C-19): sc-150). Just above 37 kDa and below the 37 kDa breakdown fragments of the larger human isoforms are detected.

**Figure 2:**
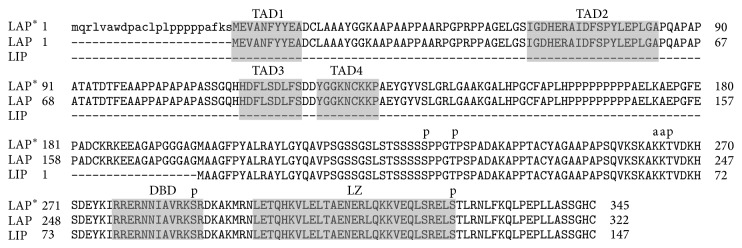
Alignment of CEBPb isoforms LAP^*^, LAP, and LIP created and annotated using reference sequence (LAP^*^: NP_005185; LAP: NP_001272807; LIP: NP_001272808) [38, 39]. Transactivation domain (TAD) 1–4, the DNA binding domain (DBD), and the leucine zipper domain (LZ) are indicated with the shaded boxes. Phosphorylation sites are indicated using the letter “p” and acetylation sites with the letter “a.”

C/EBP-*β* is an intronless gene that codes for the production of a single mRNA [16, 35]. The mouse C/EBP-*β* mRNA transcript can translate into four different protein isoforms: full length C/EBP-*β* or LAP^*^ (liver-enriched transcriptional activating protein star) with an atomic mass of 38 kDa, LAP (liver-enriched transcriptional activating protein), which has an atomic mass of 35 kDa, the 20 kDa isoform LIP (liver-enriched transcriptional inhibitory protein), and a smaller 16 kDa isoform (Figure 1). The isoform LAP is a transcriptional activator, while the isoform LIP, which lacks the transactivation domain, is a transcriptional inhibitor (Figure 2). This results in isoform specific transcriptional activation potential [16, 36, 37]. Already in 1991, Descombes and Schibler [35] have shown that the isoforms LIP and LAP have antagonistic activities. Also in heterodimerized form with other family members, LIP inhibits the transcriptional activation activity of its partner [9, 36]. Together, these data suggest that LIP acts as a dominant negative regulator of other C/EBP family members.

In some articles authors use the term “LIP/LAP ratio” to refer to changes in the amount of LAP^*^, LAP, or LIP protein that is produced. In our perception, one should also refer to exact concentrations of LAP^*^, LAP, or LIP, since a ratio does not give information on the amount of each isoform that is produced. For example, there is a difference between a LIP/LAP ratio, for example, 8/2 or 100/25, while both ratios seem equal: 4. At high amounts of the transcription factors the biological effects are likely to differ when compared to lower concentrations, for example, at high dose (one of) the heterodimeric partners of LIP/LAP might become limiting or all binding places might become fully occupied.

## 3. Transcription of C/EBP-**β**

### 3.1. Activation of C/EBP-*β* Transcription

In the C/EBP-*β* promoter various binding sites allow binding of transcription factors that directly influence transcription of C/EBP-*β* mRNA (Table 1). Furthermore, there are two cAMP-like responsive elements (CRE-like sites) in the region close to the TATA box of the C/EBP-*β* gene. The PKA/CREB pathway targets these two CRE-binding sites and thereby regulates the transcription of C/EBP-*β* [40]. In addition, C/EBP-*β* is able to stimulate its own transcription [40].

### 3.2. Isoform Specific Translation

A possible model to explain the production of the various C/EBP-*β* isoforms involves a “leaky ribosome scanning mechanism” [72]. In this model, the first AUG codon is ignored by the ribosomes that are scanning the C/EBP-*β* mRNA, resulting in translation initiation starting from the next AUG codon (Figure 2) [72]. As an alternative, Timchenko et al. [73] proposed another pathway for LIP production named “proteolytic cleavage,” which is regulated by another member of the C/EBP family; C/EBP-*α* [74]. Since low proteolytic activity was found in cultured cells, they also concluded that the generation of LIP is predominantly depending on translational regulation [73].

## 4. C/EBP-**β** Target Genes

Although C/EBP-*β* increases the expression of a wide variety of target genes that regulate numerous metabolic processes (Table 1), C/EBP-*β* binding sites are particularly found in regulatory sequences of genes that are associated with, that is, the inflammatory response [21], or the ER stress pathway [75]. In addition, several C/EBP-*β* protein interactions and regulatory factors that are involved in C/EBP-*β* transcription have been reported (Table 1).

## 5. The Role of C/EBP-**β** in Metabolic Regulation

**Figure 3:**
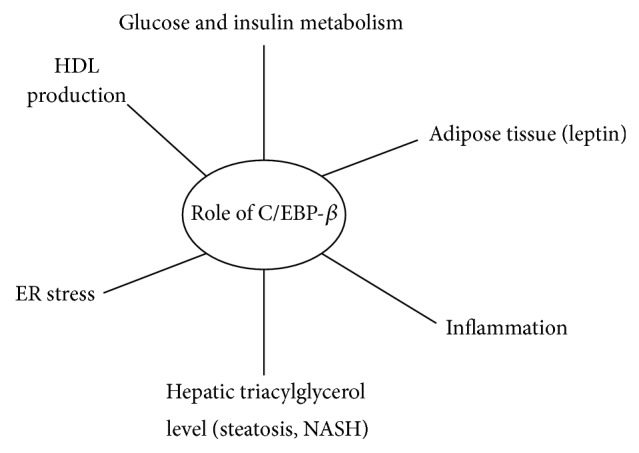
Simplified scheme of the involvement of C/EBP-*β* in factors related to the metabolic syndrome as described in literature.

Numerous studies suggest a role for C/EBP-*β* in pathways related to the metabolic syndrome. Current insights regarding the involvement of C/EBP-*β* in adipose tissue differentiation, glucose and insulin metabolism, triacylglycerol metabolism, hepatic steatosis, endoplasmic reticulum stress, inflammation, and HDL production will be described in the following sections (Figure 3). When discussing the influence of C/EBP-*β* on metabolic aberrations, it is important to consider the effects of C/EBP-*β* on weight or adipose tissue loss, as weight loss might consequently induce positive effects on the features of the metabolic syndrome [76, 77].

### 5.1. The Role of C/EBP-*β* in Adipose Tissue Mass and Adipocyte Differentiation

C/EBP-*β*, as well as other members of the C/EBP family, plays a role in adipocyte differentiation and maturation, suggesting involvement in the etiology of overweight and obesity. When wild type and C/EBP-*β* deficient mice were fed a high-fat diet (60 en%) for 12 weeks, the wild type mice gained weight, while the knockouts did not and even lost body fat. Also on a low-fat diet, the C/EBP-*β* knockouts had less total body fat [13]. Similar findings were reported by Staiger et al. [78]. Although we can certainly not rule out that weight loss itself contributed considerably to the observed healthier metabolic profile, the C/EBP-*β* deletion resulted in decreased expression of hepatic lipogenic genes and lowered expression of acetyl-CoA carboxylase and reduced fatty acid synthase, suggestive for a reduced hepatic fatty acid production and increased lipolysis [13]. In addition, after the high-fat diet, energy expenditure, which was measured by CO_2_ production, was increased in the C/EBP-*β* knockouts, while the amount of brown adipose tissue was not increased. However, although brown adipose tissue was not elevated, the explanation for the increased energy expenditure in the C/EBP-*β* knockout mice was explained by elevated gene expression in brown adipose tissue *β*-oxidation (LCAD and AOX). Although UCP1 and UCP3 in the muscle were clearly increased, the change in UCP expression in BAT did not reach statistical significance [13].

C/EBP-*β* and C/EBP-*α* are postulated as transcriptional activators for the mouse UCP gene, as two binding places for C/EBP were detected in the UCP gene promoter. Cotransfection of the UCP-CAT vector with C/EBP-*β* resulted in increased transactivation of the UCP promoter in primary brown adipocytes of rats [79]. Furthermore, when exposed to cold, which is a stimulus for development of brown fat and UCP expression, specifically C/EBP-*β* expression showed a time dependent increase in brown adipose tissue of adult rats. Here, LIP protein production increased rapidly after 12 to 24 hours of cold exposure. Additionally, C/EBP-*β* mRNA expression and LIP protein production was higher during fetal development of brown adipose tissue compared to adult brown adipose tissue and during BAT development in the fetus the amount of LIP decreased gradually [80]. These data suggest a role for C/EBP-*β* and particularly its isoform LIP in the (fetal to adult) development of brown adipose tissue BAT and in increasing brown adipose tissue activity.

As described in Table 1, C/EBP-*β* can induce both PPAR-*γ* and C/EBP-*α* gene expression, since both genes contain C/EBP binding sites in their proximal promoters [67, 68]. Particularly, during the first two days of white adipocyte differentiation, C/EBP-*β* and C/EBP-*δ* levels are increased, after which levels decrease sharply before C/EBP-*α* levels increase [16]. During the early stage of adipogenesis, C/EBP-*β* and C/EBP-*δ* mRNA activate transcription of PPAR-*γ* [81]. Mice deprived of the C/EBP-*β* gene did show white adipocyte development; for example, they could not store lipids inside the adipocytes, regardless of the presence of C/EBP-*α* and PPAR-*γ* [81]. In addition, Chung et al., 2012 [82], suggested that activation of Wnt-*β* inhibits activations of PPAR-*γ* and C/EBP-*α* that are controlled by C/EBP-*β*. When adipogenic inducers (such as insulin) were added, knockdown of C/EBP-*β* inhibited adipogenesis, while activated signaling of Wnt-*β* was maintained. When the C/EBP-*β* gene was overexpressed, signaling of Wnt-*β* was inhibited. These findings suggest that C/EBP-*β* can inhibit Wnt-*β* signaling and that C/EBP-*β* is necessary to stimulate the expression of genes responsible for adipogenesis [82]. Zuo et al. have also evaluated the role of C/EBP-*β* in the activation of C/EBP-*α* [67]. In an earlier study it was shown that after inhibition of C/EBP-*β* activity by ectopic expression of the protein LIP, the expressions of C/EBP-*α* and PPAR-*γ* were blocked [83]. This suggests that C/EBP-*β* isoform LIP modulates the expression of C/EBP-*α*. In the past, the role of C/EBP-*β* in inducing adipogenesis via PPAR-*γ* was already extensively investigated [84, 85]. For example, the truncated isoform LIP inhibited adipocyte differentiation in 3T3-L1 cells [84]. However, these studies could not examine effects on C/EBP-*α* induction, since NIH-3T3 fibroblasts were used, which do not produce C/EBP-*α* [67, 86]. Zuo et al. now suggested that C/EBP-*β* is able to activate adipogenesis through the stimulation of PPAR-*γ*, which activates C/EBP-*α* expression [67].

The role of the adipokine leptin as a mediator of energy balance is well known in humans [87, 88]. ChIP analysis suggested that leptin levels are decreased by C/EBP-*β* via association with the promoter of leptin. This finding is confirmed with observations showing that the leptin promoter contains a C/EBP motif binding site [20], to which C/EBP-*α* can bind [89]. In C/EBP-*β* knockout mice a reduction in leptin but also a decrease in fat mass was observed [78]. However, as leptin is produced by white adipose tissue, it is also possible that the observed decrease in leptin simply results from the decrease in the amount of body fat.

In summary, in vitro and animal studies suggest that C/EBP-*β* plays an important role in promoting the development and differentiation of both white and brown adipose tissue. In addition, C/EBP-*β* plays a role in increasing brown adipose tissue activity, via elevated UCP expression in brown adipose tissue. Furthermore, the C/EBP family might be involved in the production of leptin.

### 5.2. The Role of C/EBP-*β* in Glucose and Insulin Metabolism

**Figure 4:**
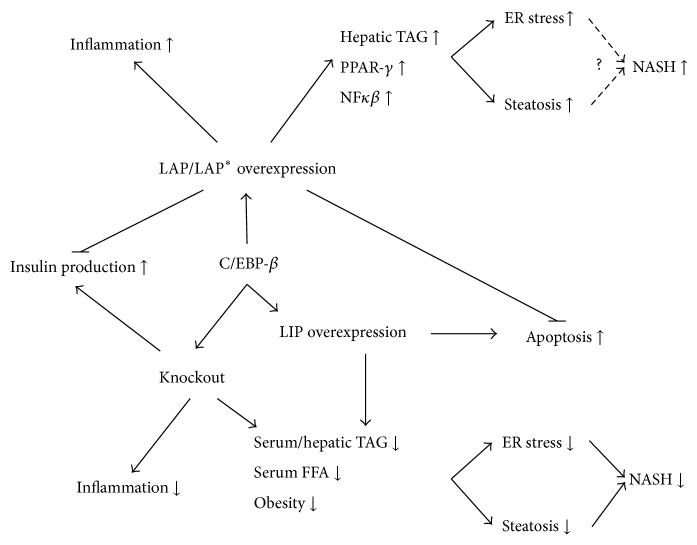
C/EBP-*β* in metabolic processes related to the metabolic syndrome.

Besides a potential indirect effect of C/EBP-*β* on metabolism via a reduced adipocyte mass, there are indications that C/EBP-*β* directly affects glucose and insulin metabolism (Figure 4). A decreased hepatic C/EBP-*β* expression coincides with increased insulin production [20, 90, 91]. Rats and mice fed a high-carbohydrate diet (81% sucrose), thereby increasing insulin secretion 5-fold, showed an 80% decreased hepatic C/EBP-*β* mRNA production compared to animals fed a standard diet (41% starch) [90]. Matsuda et al. found that pancreatic *β*-cell specific C/EBP-*β* knockout mice were characterized by increased insulin secretion, while they did not differ in body weight compared to controls [92]. Furthermore, C/EBP-*β* expression was increased in hepatocytes of (streptozotocin-treated) type I diabetic rats [90]. However, when these animals were treated with insulin or with the insulin mimetic vanadate, C/EBP-*β* expression decreased again [90]. Altogether, these findings not only indicate that C/EBP-*β* plays a role in regulating pancreatic insulin secretion, but also that insulin relates to lower hepatic expression of C/EBP-*β* [90]. These effects may also translate into differences in glucose metabolism. Fifty percent of all C/EBP-*β* knockout mice die early due to disturbances in glycogen mobilization and consequent hypoglycemia [93]. After an 18 hour fast, surviving mice clearly suffered from severe hypoglycemia, decreased hepatic glucose production (40% reduction) and low plasma free fatty acid (FFA) concentrations compared to wild type mice. However, plasma insulin levels were comparable between the knockout and wild type mice. After correction for the amount of DNA per gram of adipose tissue the overnight fasted knockouts showed reduced lipid content per cell. The authors state that the decrease in fat mass might have accounted for the decreased FFA concentrations in C/EBP-*β* knockout mice. Moreover, hepatic cyclic adenosine monophosphate (cAMP) was reduced in C/EBP-*β* knockout mice during basal and glucagon stimulated conditions [93], and administration of cAMP increased glycogen mobilization, resulting in normal blood glucose concentrations [93, 94]. cAMP is an important contributor to whole-body glucose homeostasis, as it is involved in the insulin-signaling cascade by activating PKA [95]. In relation to this, it has been shown that C/EBP-*β* is required in maintaining appropriate cAMP concentrations in liver and adipose tissue [94].

Besides an effect on glucose homeostasis via affecting insulin and glucagon directly, the C/EBP gene family may also affect the uptake of glucose, as the promoter of the GLUT-4 gene contains a C/EBP binding site [96]. This might explain partly why adipocytes of C/EBP-*β* and C/EBP-*δ* deficient mouse embryonic fibroblasts (MEFs) have reduced GLUT-4 mRNA expression [97]. In addition, the insulin receptor substrate-2 (IRS-2) was decreased in these mice compared to their control littermates, which could be another explanation for reduced GLUT-4 expression. Furthermore, in HepG2 cells, transcription of the human insulin receptor (IR) is controlled by a transcriptionally active multi-protein-DNA complex. This complex is composed of nuclear protein HMGI-Y, transcription factors Sp1, and C/EBP-*β* [52]. Although IR expression was not changed in adipocytes of C/EBP-*β*/*δ* deficient mouse MEFs [97], the findings of Foti et al. suggest that C/EBP-*β* is involved in a transcriptional network needed for the transcription of human insulin receptors [52]. In contrast, deleting C/EBP-*β* in mice increased IRS-1 levels as well as skeletal muscle insulin sensitivity [98]. Moreover, despite the decreased amount of adipose tissue, which could explain (part of) the favourable metabolic effects, mice with a C/EBP-*β* gene deletion showed reduced plasma free fatty acid concentrations and increased insulin-signal transduction in skeletal muscle, indicating improved whole-body insulin sensitivity [98]. This was supported by findings that C/EBP-*β* suppression in palmitate-treated 3T3L1 cells improved Akt phosphorylation in response to insulin [99]. Furthermore, in mice, the accumulation of C/EBP-*β* leads to failure of pancreatic *β*-cells, due to increased vulnerability to ER stress. These findings suggest that C/EBP-*β* is also involved in the onset of insulin resistance and type II diabetes [92].

In conclusion, animal and cell studies suggest that C/EBP-*β* influences insulin and glucose metabolism. However, in most cases the effect of C/EBP-*β* knockout resulted in decreased body weight or adipose tissue loss, which might have caused the reduction of blood glucose and free fatty acids. Since the accumulation of C/EBP-*β* in the pancreatic *β*-cells may increase the risk for type II diabetes in animals, it might be interesting to investigate the contribution of C/EBP-*β* in the onset of type II diabetes in humans.

### 5.3. The Role of C/EBP-*β* in Triacylglycerol Metabolism and Hepatic Steatosis

Although we cannot rule out the beneficial metabolic effects of subsequent weight loss, C/EBP-*β* knockout mice showed lower serum and triacylglycerol concentrations and a decreased hepatic triacylglycerol content, when compared to their wild type littermates on the same high-fat diet (Figure 4) [13]. In addition, in Lepr(db/db) mice, where no weight loss could be detected, the deletion of C/EBP-*β* reduced hepatic fat content and thereby the risk to develop diabetes and obesity [100]. Moreover, hepatic triacylglycerol content as well as lipogenic enzyme activity of C/EBP-*β*(−/−) × Lepr(db/db) mice was dramatically decreased in comparison to wild type mice. However, in the same study, overexpression of C/EBP-*β* isoform LIP in wild type mice resulted in a 50% reduction of hepatic triacylglycerol concentrations. This might be explained by the fact that LIP is a dominant negative protein, which might inhibit other C/EBP-*β* isoforms that seem to cause steatosis [100].

Nonalcoholic steatohepatitis (NASH) is strongly associated with obesity, type II diabetes, and the metabolic syndrome [101]. The first step in development of NASH is the increased accumulation of triacylglycerol in the liver caused by lipid overflow. Next, inflammation is induced which can eventually result in development of fibrosis and ultimately liver cell death. Rahman et al. have shown that C/EBP-*β* knockout mice, in which NASH was induced using a methionine-choline deficient diet (MCDD), were partly protected from the development of steatosis, although the results on weight loss in MCDD fed C/EBP-*β* knockout mice were not shown [102]. The authors also mention the possibility of C/EBP-*β* deletion leading to reduced accumulation of lipids in the liver. They ascribe the decreased steatosis development to decreased lipogenesis, resulting in decreased hepatic triacylglycerol content and a decreased activation of inflammation [102]. Similar to the phenotypic response in MCDD fed mice, C/EBP-*β* overexpression in hepatocytes of wild-type mice increased PPAR-*γ* activation, NF*κ*B, hepatic triacylglycerol level, steatosis, and ER stress. These data suggest that high C/EBP-*β* levels contribute to the development of NASH and that C/EBP-*β* inhibition is potentially beneficial in preventing hepatic steatosis.

### 5.4. The Role of C/EBP-*β* in Endoplasmic Reticulum Stress

The endoplasmic reticulum (ER) plays a role in folding newly synthesized proteins [103]. In conditions of ER stress, poorly folded proteins accumulate in the ER, which is detected by the three main ER stress sensors IRE1*α*/*β*, PERK, and ATF6 [104–106]. The master regulator of ER stress is GRP78 or BiP protein. When GRP78 detects ER stress, it dissociates from the ER stress sensors to activate the unfolded protein response (UPR) [104]. Initially, the UPR copes with ER stress by introducing chaperones and by attenuation of protein translation. However, persistent ER stress will eventually trigger cell death or apoptosis [105].

In cultured HepG2 cells, C6 cells, and mouse insulinoma cells, C/EBP-*β* (especially the C/EBP-*β* isoform LAP) was activated during ER stress (Figure 4) [55, 75, 107]. This increased LAP production was followed in time by an increase of the isoform LIP. Meir et al. have shown that LAP overexpression decreased, whereas LIP overexpression increased ER stress triggered cell death [75]. These findings were confirmed by Li et al. who furthermore showed that C/EBP-*β* binds to ATF4 and CHOP (also named C/EBP-*ζ*), which are both induced during the UPR [107]. LIP lowered the expression of prosurvival ATF-4 target genes, and C/EBP-*β* was found to increase the production of CHOP and its downstream cell death related proteins [107]. Besides hepatocytes, also other tissues such as adipocytes, macrophages, and *β*-cells are targeted by C/EBP-*β* induced ER stress. For example, Matsuda et al. showed that accumulation of C/EBP-*β* in the *β*-cells of the pancreas of diabetic mice induced loss of *β*-cell mass and insulin production [92]. The explanation for this finding was the accumulation of C/EBP-*β* blocking ATF6-mediated GRP78 transcription, which makes cells more vulnerable for ER stress and ultimately to the onset of type II diabetes [92].

Together, these results show a link between C/EBP-*β* and ER stress. C/EBP-*β* isoform LIP appears important in the switch from a protective to an apoptotic pathway in cells that are exposed to ER stress related UPR. This makes C/EBP-*β* and its isoforms interesting for further research in the prevention of ER stress in humans, in which reduction of isoform LIP seems beneficial to reduce ER stress triggered cell death.

### 5.5. The Role of C/EBP-*β* in the Inflammatory Cascade

Evidence for a role of the different members of the C/EBP family in the inflammatory response rapidly increases [99, 108]. Many studies have shown that C/EBP-*β* is transcriptionally activated by inflammatory stimuli such as turpentine oil, cytokines such as IL-6, IL-1, and TNF-*α*, and bacterial LPS [108]. When the transactivation domain of C/EBP-*β* becomes phosphorylated due to the presence of inflammatory stimuli, transcription of the C/EBP-*β* gene increases [109]. C/EBP-*β* on its turn elevates expression of various proinflammatory genes. It is generally accepted that C/EBP-*β* is a key regulator of IL-6 signaling and is important in transcriptional regulation of the IL-6 gene [31, 110]. IL-6 is a key player in various characteristics of the metabolic syndrome, since it is an important contributor to the low-grade proinflammatory state [111]. IL-6 on the other hand suppresses the production of insulin in subjects with type II diabetes, which indicates increased insulin sensitivity [112]. One question is whether all C/EBP-*β* isoforms are equally important in its effects on inflammation. After treatment of BALB/c mice with LPS, particularly the expression of the LIP isoform was strongly increased whereas the expression of isoform LAP did not change [113]. This could indicate that particularly an increase in isoform LIP is important in regulating the inflammatory state.

In macrophages, C/EBP-*β* is involved in coordinating the expression of IL-1, IL-6, IL-8, TNF-*α*, granulocyte colony-stimulating factor (G-CSF), nitric oxide synthase, neutrophil elastase, myeloperoxidase, and lysozyme and the macrophage granulocyte and granulocyte-macrophage receptor genes [114]. C/EBP-*β* is also able to increase the gene expression of macrophage inflammatory protein 1 (MIP-1*α*), osteopontin, and CD14 in a monocytic cell line (M1 cells) [114]. In addition, C/EBP-*β* knockout mice showed an impaired ability to activate macrophages, pointing towards a distorted immune response [94]. Moreover, C/EBP-*β* knockout mice suffered from defects in their innate, humoral, and cellular immunity, which is due to a deficiency in the activation of splenic macrophages, an impaired IL-12 production (involved in activation of natural killer cells and T-cells), and an altered T-helper function. These data reveal that C/EBP-*β* is crucial for the accurate functioning of the immune response, in particular of haemopoietic and lymphoid compartments. Further, Yan et al. showed in macrophages that C/EBP-*β* and C/EBP-*δ* activated the inflammatory response even more when overexpressed together, suggesting that C/EBP-*β*-C/EBP-*δ* heterodimers are more potent activators [115].

There is also a link between C/EBP-*β* expression and inflammation in high-fat treated RAW 264.7 macrophage cells, 3T3-L1 adipocytes [99]. C/EBP-*β* deletion completely blunted the high-fat diet-induced development of inflammation [99]. Moreover, IL-10 and LXR*α* gene expression as well as its targets (SCD1 and DGAT2) was largely increased in peritoneal C/EBP-*β* knockout macrophages. Even more, they showed suppressed expression of the NLRP3 gene, which is necessary for the activation of the inflammasome [99]. In the macrophage cell line RAW 264.7 or in 3T3-L1 adipocytes, knockdown of C/EBP-*β* also blocked the onset of inflammation after palmitate addition, probably via a decreased activation of p65-NF*κ*B [99]. The latter finding was confirmed by performing a C/EBP-*β* overexpression experiment in which NF*κ*B binding, proinflammatory cytokine gene expression, and JNK activation were indeed increased [99]. Finally, Screpanti et al. showed diminished NO production after* C. albicans* infection by macrophages from C/EBP-*β* knockout mice, while wild type macrophages were perfectly capable of producing the vasodilator NO [31].

These results suggest that C/EBP-*β* is an important contributor in the onset of the inflammatory response. It would be interesting to evaluate the effects of C/EBP-*β* inhibition in the prevention of obesity induced systemic inflammation. Evaluation of the most important isoform within this context also deserves attention.

### 5.6. The Role of C/EBP-*β* in HDL Metabolism

Large-scale epidemiological studies suggest that increased high-density lipoprotein cholesterol (HDL-C) concentrations protect against the development of cardiovascular diseases [116, 117]. However, recent studies failed to show that an increase in serum HDL-C levels translates into a lower CVD risk [118]. Nowadays, the emphasis is on increasing HDL functionality [119] and there is growing evidence that the protective effects of HDL-C depend on apoA-I, the main protein constituent of an HDL particle [120, 121]. The apoA-I promoter has a C/EBP binding site, which suggests C/EBP-*β* could be involved in the production of apoA-I. However, available data for a possible role of C/EBP-*β* in regulating apoA-I production is not conclusive. Although Kan and colleagues [122] concluded that C/EBP-*β* was not involved in apoA-I production, this was not explored during inflammatory conditions. Testing this hypothesis during inflammatory conditions might be interesting since inflammation is a prominent feature of the metabolic syndrome, and apoA-I is a negative acute phase protein. Moreover, effects of different isoforms were not explored by Kan. Given the potential differences in regulatory effects, it is possible that specific C/EBP-*β* isoforms influence apoA-I production differently.

## 6. Conclusions 

We have evaluated a possible role for C/EBP-*β* and its isoforms in the etiology and progression of the metabolic syndrome (Table 2 and Figure 4). Currently, all data available regarding the role of C/EBP-*β* arise from animal and in vitro experiments whereas data from human studies is lacking. There is evidence that C/EBP-*β*, in particular its isoform LIP, plays a role in the development of white and brown adipose tissue and in increase activity of brown adipose tissue. Furthermore, animal studies showed that C/EBP-*β* knockout results in weight loss, lower plasma free fatty acids, and decreased plasma glucose concentrations. However, one should be aware that C/EBP-*β* deletion coincides with a strong reduction in body weight and fat mass. This decline in fat mass can be ascribed to the prominent role of C/EBP-*β* in adipogenesis. Therefore, it is questionable whether the metabolic effects described are due to C/EBP-*β* itself or are actually indirect effects due to an inability to increase in body weight and in fat mass. Besides these metabolic effects there is a vast amount of evidence showing a role of C/EBP-*β* in increased inflammatory response and ER stress. In conclusion, in light of these results, it is also important to examine the potential role of C/EBP-*β* in humans with and without the metabolic syndrome.

## Figures and Tables

**Table 1:** Regulatory factors for C/EBP-*β* production, C/EBP-*β* target genes, and C/EBP-*β* protein interactions (please note that this list is not exhaustive, for a more extensive list also visit the GenCards website [41]).

Regulatory factors in C/EBP-*β* transcription	C/EBP-*β* target genes	C/EBP-*β* protein interactions
Sp1 [42]	IL-6 [43]	CREB1 [44]
CREB/ATF [42]	TNF-*α* [21]	CRSP3 [45]
SREBP1c [46]	IL1-*β* [21]	DDIT3/CHOP [47]
RARa [48]	IL-8 [49]	EP300 [50]
Myb [51]	IL-12 [21]	HMG-I/HMG-Y [52]
Fra-2 [53]	G-CSF or CSF3 [21]	HSF-1 [54]
EGR2 or KROX20 [55]	Receptors for G-CSF, GM-CSF, M-CSF [21]	SWI/SNF complex [56]
STAT-3 [57]	MIP1-*α* [43]	Sp1 [52]
NFkB [58]	Osteopontin [43]	TRIM28/KAP1 [59]
C/EBP-*β* [40]	CD14 [43]	EGR-1/zif268/NGFI-A [60]
	MIP1-*β* [15]	Smad-3 and Smad-4 [61]
	CRP [21]	ATF2 [62]
	Hemopexin [21]	ATF4 [63]
	Haptoglobin [15]	C/EBP-*α*, *β*, *γ*, *δ*, *ζ* [9]
	AGP-a1 [21]	FKHR [64]
	NFkB, P50 subunit [58]	
	NR3C1 [65]	
	C-FOS [66]	
	PPAR-*γ* [67, 68]	
	C/EBP-*α*, *β*, *γ*, *δ*, *ζ* [9]	
	cAMP [69]	
	Albumin [70]	
	MDR1 [71]	

**Table 2:** The involvement of C/EBP-*β* in metabolic processes.

Metabolic process	Involvement of C/EBP-*β*	C/EBP-*β* knockout	C/EBP-*β* overexpression
Adipose tissue development, white and brown	White adipocyte differentiation and maturation (also role for C/EBP-*α*)	Decreased body fat content	
	Brown adipocyte activity and development	Elevated gene expression in brown adipose tissue *β*-oxidation	

Leptin production	Binding possibility on leptin promoter	Decreased leptin production (possibly fat mass related)	

Glucose and insulin metabolism	High insulin = low C/EBP-*β*	Increased insulin production after hepatic knockdown	
	Low C/EBP-*β* = High insulin	Unchanged insulin production after C/EBP-*β* deletion	
	Accumulation of C/EBP-*β* induces diabetes via ER stress induction	Increased insulin sensitivity	
	Maintenance of plasma glucose levels	Hypoglycemia	
	C/EBP binding site in GLUT-4 promoter	Decreased hepatic glucose production	
		Decreased cAMP	

Triacylglycerol metabolism	Influencing lipogenic enzyme activity	Reduced plasma free fatty acid concentrations	
Hepatic steatosis-NASH	Influencing the amount of hepatic TAG	Decreased lipogenic enzyme activity	
		Decreased hepatic TAG	
		Protected from steatosis, decreased NASH development	Increased steatosis

ER stress	Accumulation of C/EBP-*β* induces diabetes via ER stress		LIP isoform increased cell death
	C/EBP-*β* is increased during ER stress		LAP isoform decreased cell death
	High LIP promotes cell death		
	LIP lowers prosurvival ATF-4 targets		
	In early ER stress response LAP production higher		
	In early ER stress response LIP production higher		

Inflammation	C/EBP-*β* activates inflammatory response	Defects in immune response, impaired macrophage activation	Activation of the immune response
	LIP isoform induces inflammation	Decreased high fat induced inflammation	

HDL particle production	C/EBP-*β* has binding place in apoA-I promoter		C/EBP no central role in expression of the apoA-I gene
